# Age-Dependent Changes in Intrinsic Neuronal Excitability in Subiculum after Status Epilepticus

**DOI:** 10.1371/journal.pone.0119411

**Published:** 2015-03-16

**Authors:** Sungkwon Chung, Nelson Spruston, Sookyong Koh

**Affiliations:** 1 Department of Physiology, Samsung Biomedical Research Institute, Sungkyunkwan University School of Medicine, Suwon, South Korea; 2 Scientific Program, Janelia Research Campus, Ashburn, Virginia, United States of America; 3 Neurobiology Program, Stanley Manne Children’s Research Institute, Northwestern University Feinberg School of Medicine, Chicago, Illinois, United States of America; Centre national de la recherche scientifique, University of Bordeaux, FRANCE

## Abstract

Kainic acid-induced status epilepticus (KA-SE) in mature rats results in the development of spontaneous recurrent seizures and a pattern of cell death resembling hippocampal sclerosis in patients with temporal lobe epilepsy. In contrast, KA-SE in young animals before postnatal day (P) 18 is less likely to cause cell death or epilepsy. To investigate whether changes in neuronal excitability occur in the subiculum after KA-SE, we examined the age-dependent effects of SE on the bursting neurons of subiculum, the major output region of the hippocampus. Patch-clamp recordings were used to monitor bursting in pyramidal neurons in the subiculum of rat hippocampal slices. Neurons were studied either one or 2-3 weeks following injection of KA or saline (control) in immature (P15) or more mature (P30) rats, which differ in their sensitivity to KA as well as the long-term sequelae of the KA-SE. A significantly greater proportion of subicular pyramidal neurons from P15 rats were strong-bursting neurons and showed increased frequency-dependent bursting compared to P30 animals. Frequency-dependent burst firing was enhanced in P30, but not in P15 rats following KA-SE. The enhancement of bursting induced by KA-SE in more mature rats suggests that the frequency-dependent limitation of repetitive burst firing, which normally occurs in the subiculum, is compromised following SE. These changes could facilitate the initiation of spontaneous recurrent seizures or their spread from the hippocampus to other parts of the brain.

## Introduction

Kainic acid (KA)- induced seizures are widely used as an experimental model of temporal lobe epilepsy, the most common surgically remediable drug-resistant epilepsy syndrome [1]. A single systemic injection of KA in mature rodents results in status epilepticus(SE), and following a period of about two weeks of no obvious behavioral motor seizures, animals develop epilepsy characterized by spontaneous recurrent seizures [2–4]. Neural processes that occur during this so- called latent period, defined as the time between the initial insult and first occurrence of a convulsive seizure, include cell death, cell birth, axonal sprouting and electrophysiological changes [4, 5]. These processes contribute to epileptogenesis, but may be separate from the acute molecular and cellular changes caused by seizures [6]. For example, KA induced status epilepticus (KA-SE) results in a pattern of hippocampal neurodegeneration that resembles human hippocampal sclerosis [5, 7–10], but significant neuronal death following status epilepticus is not required for later spontaneous seizures to occur [11, 12].

A characteristic of several animal models of epilepsy is that immature rats younger than postnatal day (P) 18 are far less likely to develop spontaneous recurrent seizures or cell death compared to adult rats [2, 9, 13, 14]. Rather, they exhibit a limited pathology characterized by a lowered seizure threshold [15–21]. Studying the age-dependent changes following status epilepticus, therefore, offers a strategy for understanding epileptogenesis and for identifying factors that contribute to seizure susceptibility.

In this study, we examined whether changes in neural excitability occur in the subiculum of KA-treated immature (P15) and mature (P30) rats. We studied the subiculum because it constitutes the major output region of the hippocampus, heavily connected with several regions, including neocortex [22–24]. Many studies have demonstrated KA-induced changes in other areas of the hippocampus [5, 7], but neural changes in the subiculum would facilitate the spread of hippocampal hyper-excitability to other brain regions, a necessary condition for the development of generalized convulsions. Furthermore, pyramidal cells in the subiculum exhibit variable and self-limited bursting [25–28]. Upregulation of bursting and/or downregulation of the transition from bursting to regular spiking would amplify the output of the hippocampus [28], thus increasing the likelihood of seizure spread. We have observed such changes in responses to elevated synaptic activity in rat hippocampal slices [29, 30] and upregulation of calcium currents contributing to bursting have been observed in adult rats experiencing SE [31]. Finally, recordings from humans and in human hippocampal slices prepared from patients with advanced mesial temporal lobe epilepsy revealed that activity resembling inter-ictal events could be generated in subiculum [29, 32], suggesting that pathology is likely to develop in this area.

## Materials and Methods

### Experimental Design

**Fig 1:**
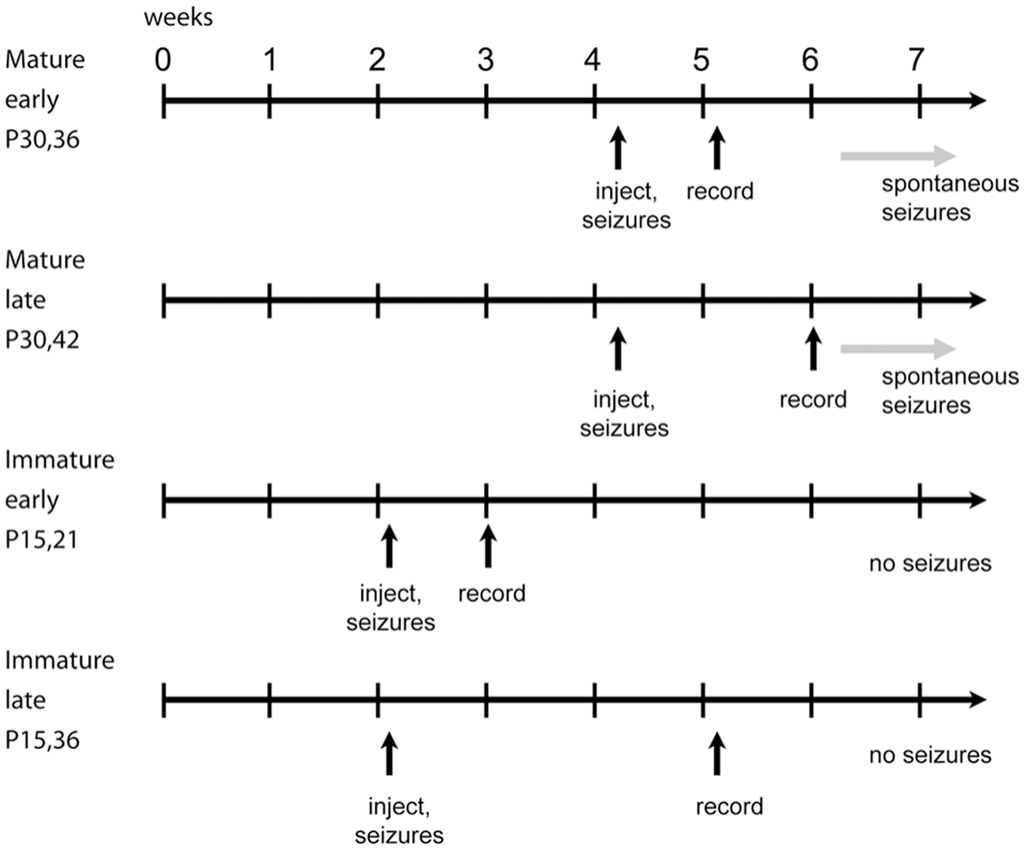
Timeline for studying the effects of KA-SE. Relatively mature (30 day-old) and immature (15 day-old) rats were injected with either saline (control) or KA. About 80% of rats injected with KA experienced seizures for over 1 hr after the injections; only these animals that experienced status epilepticus were used for all subsequent experiments. For P30 rats injected with saline or KA, slices were prepared either 5–7 days after injection (P30,36 control; P30,36 KA) or 12–13 days after injection (P30,42 control; P30,42 KA). For P15 rats injected with saline or KA, hippocampal slices were made 5–7 days after injection (P15,21 control; P15,21 KA) or 20–22 days after injection (P15,36 control; P15,36 KA).

Systemic KA was used to induce SE in P15 or P30 animals. Recordings were made either 5–7 days after injection (early) or 11–13 or 20–22 days after injection (late). The early and late time points were chosen to monitor the progression of electrophysiological changes caused by KA-SE. The early group was designed to determine whether electrophysiological changes occur soon (5–7 days) after the initial KA-SE for both P15 and P30 rats. The late group for P30 rats was designed to determine whether changes occur later (11–13 days), but still prior to the development of spontaneous behavioral seizures. Each group is denoted by an injection age and a recording age and a condition (control or KA). Four groups of animals were therefore considered (P15,21; P15,36; P30,36; P30,42) and for each group we compared animals injected with saline or KA. Timeline for studying the effects of KA-SE is shown in Fig. 1. The numbers of animals in each group are indicated in Table 1.

**Table 1:** Electrophysiological properties of subicular neurons.

	P15,21 control	P15,21 kainate	P15,36 control	P15,36 kainate	P30,36 control	P30,36 kainate	P30,42 control	P30,42 kainate
	(*n* = 4)	(*n* = 4)	(*n* = 3)	(*n* = 4)	(*n* = 6)	(*n* = 6)	(*n* = 3)	(*n* = 4)
***V***_***rest***_ **(mV)**	-62.6±0.5 (29)	-61.1±0.4* (20)	-64.0±0.5 (16)	-63.4±0.5 (31)	-63.9±0.5 (32)	-62.4±0.4* (40)	-63.8±0.5 (19)	-64.9±0.4 (25)
***R***_***N***_ **(MΩ)**	36.3±1.5 (24)	40.3±1.5 (22)	30.1±1.6 (16)	39.7±2.2** (27)	37.8±2.0 (22)	44.2±2.1 (35)	30.5±1.5 (15)	40.1±2.8* (18)
**Sag ratio**	0.77±0.01 (33)	0.78±0.01 (29)	0.78±0.01 (20)	0.79±0.01 (32)	0.77±0.01 (25)	0.78±0.01 (41)	0.78±0.01 (13)	0.78±0.01 (14)
**600 ms rheobase (pA)**	240±12 (30)	221±8 (20)	248±20 (16)	228±11 (32)	262±11 (32)	223±11** (40)	248±17 (18)	211±12* (26)
**600 ms rheobase (mV)**	-46.7±0.4 (34)	-46.4±0.5 (29)	-46.5±0.6 (20)	-46.2±0.4 (33)	-45.8±0.5 (30)	-45.3±0.5 (44)	-45.2±1.1 (6)	-46.5±0.5 (16)
**AP amplitude (mV)**	95.3±1.1 (34)	97.4±1.6 (29)	104±2 (20)	98.7±1.6* (33)	94.2±1.4 (30)	96.4±1.4 (44)	95.7±1.8 (6)	100±1.8 (16)
**AP half-width (ms)**	1.09±0.03 (33)	1.00±0.05 (29)	0.90±0.04 (9)	1.00±0.03 (33)	1.06±0.04 (30)	0.99±0.03 (44)	0.95±0.04 (6)	0.97±0.03 (16)
**sEPSC rheobase (pA)**	1060±32 (30)	1035±23 (20)	1028±71 (16)	1033±50 (28)	1149±54 (32)	997±41* (41)	1050±51 (19)	972±60 (26)

Two-way ANOVA followed by Bonferroni post-hoc tests,

*p<0.05;

**p<0.01;

AP = action potential; *n* = animals; (cells).

**Fig 2:**
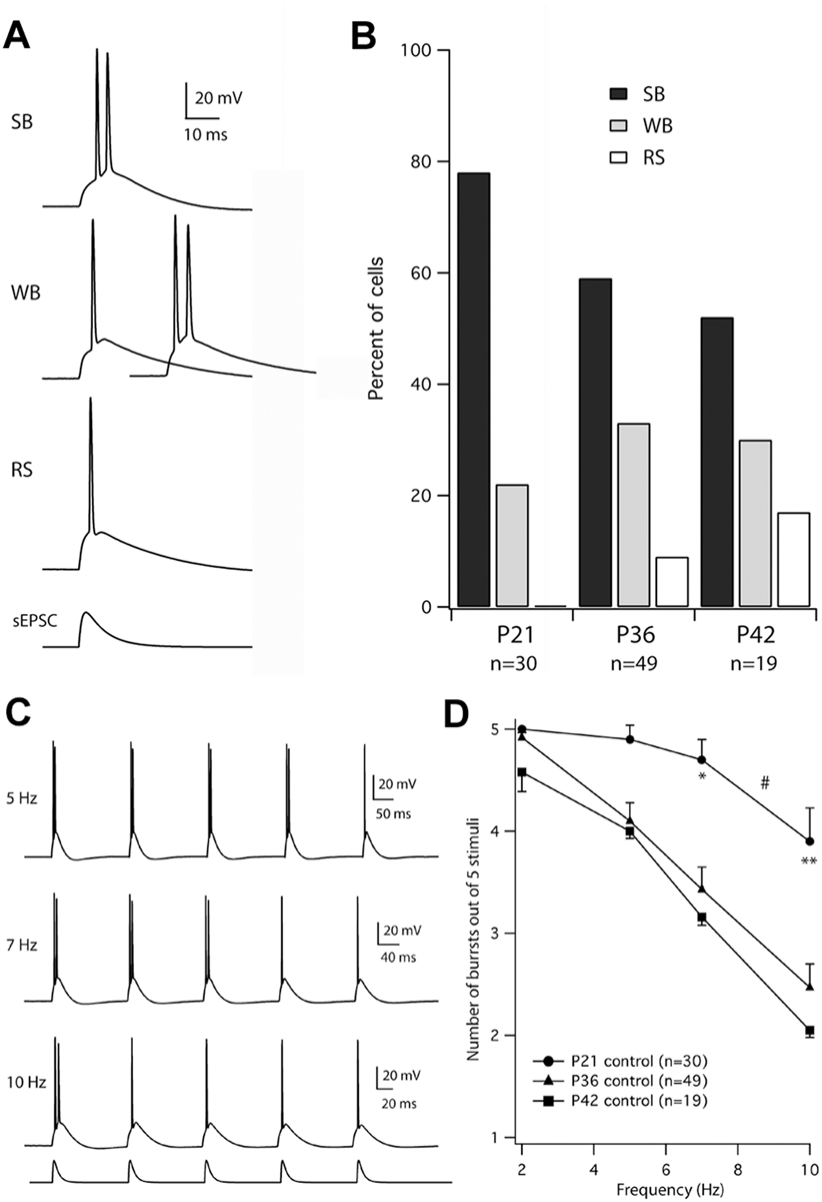
Variability and developmental changes in frequency-dependent bursting of subicular neurons. **A**. Whole-cell current-clamp recordings in response to brief sEPSCs. Top: a strong-bursting (SB) neuron exhibiting bursting in response to an sEPSCs at threshold for spiking (1.05 nA). Middle: a weak-bursting (WB) neuron exhibiting single spiking at threshold (1.25 nA) and bursting just above threshold (1.45 nA). Bottom: a regular-spiking (RS) neuron exhibiting single spiking at threshold (1.70 nA) and well above threshold (not shown). The time course of the sEPSC is shown at the bottom. **B**. The proportions of SB, WB, and RS neurons are plotted for saline-injected (control) animals (P15,21; P21,36 and P30,36 pooled; P30,42). **C**. Sample responses to sEPSC injection at 5, 7, and 10 Hz in a neuron from a P30,36 control rat. The current injection is shown schematically below the 10 Hz trace. **D**. The average number of bursts in response to five sEPSC injections delivered at 2–10 Hz decreased from the youngest (P21 control) to older (P36, control) and the oldest (P42 control) rats. ^#^Burst-frequency curves are affected by age, *p*<0.0001 (two-way ANOVA). P21 is significantly different from P36 and P42: *p<0.05 and **p<0.01 (post-hoc Bonferroni multiple comparison).

We hypothesized that epileptogenic process after KA-SE is progressing prior to development of frequent daily seizures. If epileptogenic process is progressive, then these pathologies should be most apparent in the mature, late (P30,42) group, but would be more modest in the other groups, either earlier group (P30,36) of animals and/or in younger animals (P15,21; P15,36), that are less likely to develop spontaneous, recurrent seizures [2]. The time between injection and recording at the late time point was longer (20–22 days) for P15 rats to maximize the chance of detecting changes that may evolve over time after KA at P15 even if no SE-induced changes in the frequency dependency of bursting were detected in 5–7 days in immature animals. This longer observation period also allowed the electrophysiological properties be compared to the properties of mature animals at day 36 (Fig. 2B, P36 control, *n* = 49). P15, 36 were transported to the recording site (with their KA-injected littermates) within a day after they received saline and allowed to survive for 3 weeks before recording, while P30,36 controls were transported at P30 and recordings were made within a week after the transport. Stress associated with transport may be responsible, at least in part, for an apparent difference (although not statistically significant) in electrophysiological characteristics in two groups of control neurons, P15,36 and P30,36 recorded at the same age (P36).

### Animals and KA-induced seizures

Long Evans rats (Charles River Laboratories, Cambridge, MA) were given intraperitoneal injection of saline (control) or KA on P15(3 mg/kg) or P30 (10 mg/kg). The terms “immature” and “mature” are used to denote the different age groups. Although neither group is fully developed, only the P30 animals exhibit a mature response to KA (i.e. the same as adults) in that they develop spontaneous, recurrent seizures following KA-SE [2, 11]. The doses of KA were chosen based on the age-dependent difference in threshold for KA-induced seizures. Each is the minimum effective dose to induce seizures for 1–3 hours (status epilepticus) while resulting in less than 20% lethality [2, 13, 19]. Seizures were observed for a three-hour period following KA injections and severity was assigned on a standard scale [19]. About 80% of rats injected with KA experienced seizures within 30 minutes of the injections and continued to exhibit behavioral seizures during a 3-hours observation period. Only rats with seizures that lasted over one hour (status epilepticus) were included in the study. P15 rats exhibited nearly continuous forelimb and hind limb clonus, tonic seizures and loss of balance often lying on their backs while P30 rats showed forceful clonic jerks, rearing and falling. Because we did not use anticonvulsants such as diazepam or Phenobarbital to abort seizures at a given time, the animals experienced convulsions for varying period of one to three hours. This variability in seizure duration may, at least in part, be responsible for the observed variability of bursting in subicular pyramidal neurons.

The animals were observed at least once per day and no seizure was detected prior to slice preparation. This is consistent with a previous study using over 120 hours of video monitoring over three months, that failed to detect any spontaneous seizure in rats younger than P20; spontaneous seizures first occurred in P30 rats seven weeks after systemic KA injection [2]. A recent study of continuous video EEG radiotelemetry detected the first motor seizures at the mean of 18 days after KA-SE in adult rats [4]. The lack of continuous EEG monitoring and the insufficient period of observation following the SE are a limitation of our study. We cannot, therefore, exclude the possibility that electrophysiological changes we see in the subiculum are acutely provoked by recurrent seizures.

All procedures were performed in accordance with the National Institutes of Health Guidelines for the Care and Use of Laboratory Animals and using methods approved by the Northwestern University Animal Care and Use Committee (Animal Welfare Assurance No. A3995–01).

### Tissue preparation

Hippocampal slices were prepared and maintained as described previously [26, 27]. Following halothane anesthesia, rats were perfused transcardially with ice-cold artificial cerebrospinal fluid (ACSF) and decapitated, and the brain was removed. Hippocampal slices (300μm thick) were then cut in ice-cold ACSF using an oscillating tissue slicer (Leica VT100, Nussloch, Germany). Slices were allowed to recover in a holding chamber for ~30 min at 35°C and then at room temperature. For physiological recordings, slices were transferred to a recording chamber and maintained at 33–36°C. Pyramidal neuron somata and dendrites were visualized on a fixed-stage microscope (Zeiss Axioscop 2, Oberkochen, Germany) using infrared differential interference contrast videomicroscopy and a Newvicon camera (C2400, Hamamatsu, Hamamatsu City, Japan). Whole-cell patch-clamp recordings were obtained from the soma under visual control.

### Solutions and drugs

ACSF was used for perfusion, dissection, and physiological recordings and contained (in mM) 125 NaCl, 25 glucose, 25 NaHCO_3_, 2.5 KCl, 1.25 NaH_2_PO_4_, 2 CaCl_2_, and 1 MgCl_2_ (pH 7.4, bubbled with 95% O_2_–5% CO_2_). In all experiments, kynurenic acid (2.5 mM), SR 95531 (2–4 μM), and atropine (1 μM) were added to the ACSF to block synaptic input. No spontaneous EPSPs or IPSPs were observed. The whole-cell current-clamp recording solution contained 115 mM K-gluconate, 20 mM KCl, 10 mM Na_2_-phosphocreatine, 10 mM HEPES, 2 mM Mg-ATP, 0.3 mM Na-GTP, and 0.1% biocytin (pH 7.3). Membrane potentials were not corrected for a -8-mV liquid junction potential. All drugs were obtained from Sigma (St. Louis, MO).

### Current-clamp recordings

Whole-cell, current-clamp recordings were made from the soma of subicular pyramidal neurons using a BVC-700 amplifier (Dagan, Minneapolis, MN). All recordings were obtained from neurons in relatively deep layers of subiculum (i.e. equal to or deeper than a projection of the CA1 pyramidal cell layer) and approximately midway between CA1 and presubiculum. Patch-clamp electrodes were fabricated from thick-walled borosilicate glass with resistances of 3–5 MΩ in ACSF. Input resistance (*R*_*N*_) was monitored using 600 ms current injections of-200 to +100 pA. The-200 pA responses were used to monitor the “sag ratio”—the ratio of the steady-state to the peak voltage change—which is caused by activation of the hyperpolarization-activated conductance (I_h_). Brief current injections were intended to mimic EPSCs by using a dual exponential function (τ_rise_ = 1 ms; τ_decay_ = 6 ms). Data were stored on a Macintosh computer (Apple, Sunnyvale, CA) via an ITC-16 analog-to-digital interface (Instrutech, Port Washington, NY).

### Data analysis

Data acquisition and analysis was performed using Igor Pro (Wavemetrics, Lake Oswego, OR). Input resistance was determined from the slope of regression-fits to the linear portions of the V-I plots. Statistical comparisons were performed using GraphPad Prism (San Diego, CA). Most comparisons were made using one-way or two-way analysis of variance (ANOVA) with post-hoc Bonferroni multiple comparison and frequency as a repeated measure. All experiments and data analysis were performed with the experimenter blind to whether each animal received saline or KA injection. However, the investigator was not able to be blind to immature/mature status of each animal, because of the difference in the size of the animal and the hippocampus.

## Results

To compare the age-dependent effects of KA-SE, rats were given intraperitoneal injection of saline (control) or KA on postnatal day 30 or 15 (P30, P15). The terms “mature” and “immature” are used to denote the different age groups. Recordings were made either 5–7 days after injection (early) or 11–13 or 20–22 days after injection (late). Each group is denoted by an injection age and a recording age and a condition (control or KA).

Pyramidal neurons in the subiculum of hippocampal slices were injected with brief, simulated excitatory postsynaptic currents (sEPSC) to characterize their firing patterns. At threshold for spiking (0.5–1.7 nA peak sEPSC), a range of responses was observed. Some neurons responded at threshold with bursts of two spikes. Other neurons responded at threshold with single spikes, but responded to bursts as current amplitude was increased. A third group of neurons responded only with single spikes at and well above threshold (Fig. 2A). By analogy to our previous nomenclature, we refer to these as strong-bursting, weak-bursting, and regular-spiking neurons. Strong bursters have a lower threshold for bursting and a greater ability to sustain repeated bursting [27]. Among animals injected with saline (controls), the fraction of cells in the strong-bursting group declined progressively with the age of the animal. Between P15 and P42 the fraction of strong bursting cells decreased from 78 to 52%, with a corresponding increase in the fraction of weak bursting and regular-spiking cells (Fig. 2B).

To further assess bursting in the three age groups (P21, P36, P42), subicular pyramidal neurons were injected with trains of five sEPSCs at 2–10 Hz (Fig. 2C). The peak amplitude of the sEPSC was 50 pA above threshold for bursting. As reported previously, neurons exhibited a frequency-dependent transition from bursting to regular spiking, with the transition occurring after fewer bursts at higher frequencies [25]. Using this protocol in slices from control animals, we observed a progressive decrease in bursting with maturation of the animals (Fig. 2D).

KA-SE caused a significant increase in the input resistance (*R*_*N*_) of subicular pyramidal neurons at the late time point in both age groups (Table 1: average 32% increase: two-way ANOVA, *p*<0.0001; age-matched controls, *p*<0.05, post-hoc Bonferroni multiple comparison). This change in input resistance did not appear to be due to changes in I_h_, as no change in the sag ratio was observed (Table 1; but see [33]). A representative trace to illustrate changes in membrane potential including sag in response to hyperpolarizing pulses of current, and differential action potential bursting behavior in response to depolarizing current are shown in S1 Fig.

KA-SE did not induce a consistent change in resting potential or in action potential parameters (Table 1). In keeping with the increased resistance, however, the minimum current required to evoke spiking in response to a long (600 ms) current injection (rheobase) was lower (average 11% decrease) in cells from KA-treated animals. This effect was small, but consistent across all groups and significant in the mature-animal group (Table 1: *p*<0.001, two-way ANOVA: age-matched controls, *p<0.05, post-hoc Bonferroni multiple comparison). However, there was no effect of KA-SE on the voltage threshold for action potentials, suggesting that the reduction in rheobase current is largely due to the increased input resistance. This input resistance change selectively influenced action-potential initiation in response to long current injections, as the threshold current required to evoke spikes using sEPSCs was not altered by KA-SE (Table 1).

**Fig 3:**
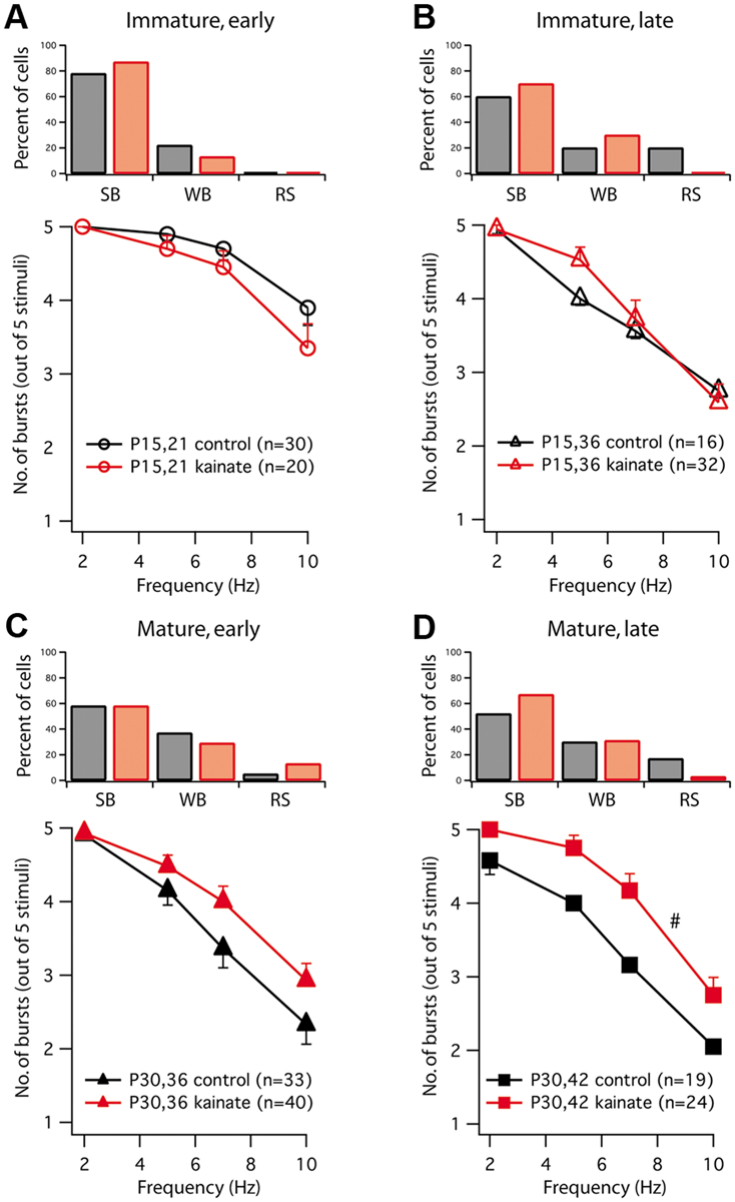
KA-SE increased bursting preferentially in mature rats. **A, B**. Top: Effects of KA on the proportions of strong-bursting (SB), weak-bursting (WB), and regular-spiking neurons in immature (P15) rats injected with saline (black) or KA (red) and measured early (**A**) or late (**B**) time point. Bottom: Effects of KA-SE on frequency-dependent bursting in the same groups of animals. **C, D**. Similar data obtained from mature animals injected with saline or KA and measured early (**C**) or late (**D**) after KA-SE. ^#^Burst-frequency curve for KA is significantly different from control, *p*<0.01 (two-way ANOVA). The total number of bursts in the mature, late group (KA versus control) is also significant when a single value (average of all cells) is compared from each injected animal, *p*<0.05 (unpaired Student’s t-test). No other curves are significantly different. The apparent appearance of difference in the proportion of cells belonging to bursting and regular spiking in two groups of control neurons, P15,36 (**B**) versus P30,36 (**C**) shown on top panels were not statistically significant (P15, 36 Control: SB + WB = 16, RS = 6; versus P30 36 Control: SB+WB = 36, RS = 2, Fisher’s exact test, *p* = 0.17).

We also examined the effects of KA-SE on bursting in subicular pyramidal neurons. In three of the four groups (except P30,36), KA-SE caused an increase in the fraction of strong-bursting neurons (Fig. 3). In P15 animals, KA-SE had no effect on the frequency dependence of bursting (Fig. 3A,B). In P30 animals, however, KA-SE caused a progressive increase in frequency-dependent bursting. At both the early and the late time points, subicular neurons from KA-injected animals exhibited more bursting in response to 5–10 Hz trains of sEPSC injections, compared to saline-injected, age-matched controls (Fig. 3C,D); however, this effect was only significant at the later time point.

## Discussion

**Fig 4:**
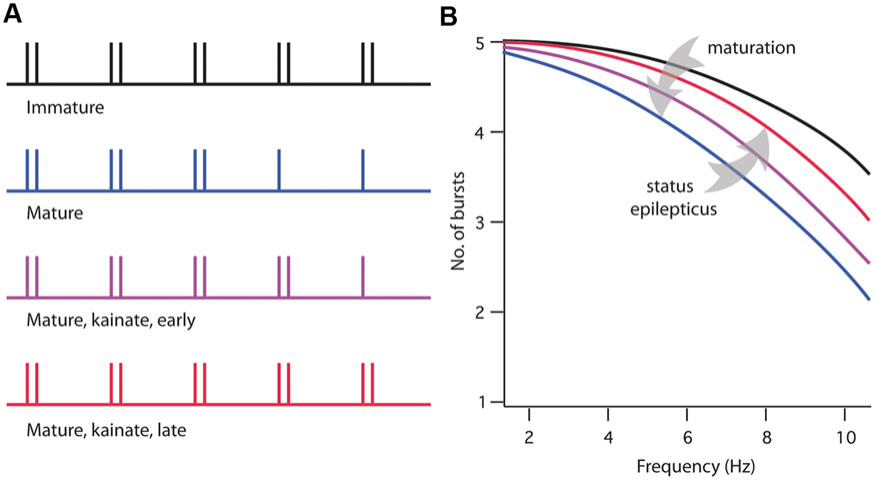
Schematic representation of developmental and KA-SE-induced changes in repetitive bursting in subiculum. **A**. Digital representation of bursting at four stages and conditions. Double and single vertical lines represent bursts and single spikes, respectively. Each train represents the response to five stimuli. **B**. Schematic plots of the number of bursts (in response to five stimuli) versus frequency at each of the stages and conditions shown. Arrows indicate the decreased bursting during maturation and the increased bursting in the latent period following status epilepticus.

We found that during postnatal maturation, between the ages of two and seven weeks, pyramidal neurons in subiculum undergo a reduction in bursting. The number of regular-spiking neurons increases from 0 to 17% during this period, with a corresponding decrease in the percentage of strong-bursting neurons. This developmental decrease in bursting was also observed in the reduced number of bursts in response to repeated current injections at 2–10 Hz. KA-SE had no effect on bursting in immature animals, but in mature animals it caused a gradual increase in bursting. These findings are summarized schematically in Fig. 4.

The changes in bursting observed following seizures in mature animals were significant two weeks after, but not one week after the KA-SE. These observations suggest that a gradual increase in bursting occurs in the weeks following SE prior to behavioral seizures [2, 11]. Thus, one plausible interpretation from our studies is that increased bursting during the latent period may contribute to the later development of epilepsy. However, because of the inadequate monitoring of spontaneous seizures, we cannot exclude the possibility that the increases in bursting we observed are secondary to acute seizures themselves; intermittent break-through seizures or subclinical seizures may occur within days in some animals after SE [4]. Another implication of our work is that the enhanced bursting we observed in immature rats could contribute to the heightened susceptibility of the developing brain to seizures [34]. Consistent with the lack of spontaneous, recurrent seizures in young animals [2], however, the post-status increase in bursting was only observed in older animals. This age-dependent effect of bursting parallels the age dependence of the development of spontaneous recurrent seizures following KA-SE.

One way of viewing the changes induced by SE is that they represent a reversion to the enhanced bursting observed at an earlier developmental state, analogous to other changes that resemble similar reversion to immature state [35–37]. Such a reversion, however, must be countered by physiologic homeostatic responses in developing animals, as younger animals with stronger bursting do not develop spontaneous recurrent seizures. Furthermore, the lack of upregulation of bursting in younger animals may reflect a ceiling effect or an inherent resistance. Thus, in parallel with the developmental reduction in bursting, other changes may render the mature brain susceptible to the network consequences of bursting. Transitions from single spiking to bursting (even with only two spikes in a burst) can dramatically increase transmitter release [38], thus leading to enhanced network activity that may eventually contribute to the development of spontaneous recurrent seizures.

The mechanisms responsible for the upregulation of bursting may be attributed to SE-induced “acquired channelopathy” [39, 40]. KA-SE may result in downregulation of K^+^ channels that regulate bursting and contribute to the increased bursting. Blocking of D-type K^+^ channels [27] with low concentrations of 4-AP, for example, enhance bursting in subicular pyramidal neurons and induce epileptic discharges in hippocampal slices [41]. KA-SE induced modification of calcium channels could also increase bursting. A high-voltage activated calcium current results in an ADP that drives bursting in subicular pyramidal neurons [26, 27]; any increase in such calcium currents could enhance bursting. On the other hand, blocking N/P/Q-type calcium channels enhances bursting due to coupling of these channels to Ca^2+-^activated K^+^ channels [26]. Modulation of prolonged inactivation of Na^+^ channels can be another mechanism by which SE could lead to enhancement of bursting. Gradual accumulation of voltage-activated sodium channels in a prolonged inactivated state can lead to frequency-dependent transition from bursting to regular spiking in subicular pyramidal neurons [25].

Our study complements several previous studies examining cellular changes in the subiculum following SE in animal models [31, 42–44]. Behr and Heinemann have shown that kindling results in a transient reduction of the post-spike AHP in subicular neurons [43]. Wellmer and colleagues observed an increase in intrinsically bursting neurons 2–5 weeks after SE induced by pilocarpine [31], suggesting that the changes are related to SE, rather than the use of KA to induce seizures. Our work builds on the work of Wellmer et al. by demonstrating that bursting increases over time. In contrast, Knopp and colleagues observed a decrease in intrinsic burst firing in subicular pyramidal neurons [44]. In that study, however, recordings were made 6–8 weeks after pilocarpine-induced SE, likely at the time when animals are experiencing frequent daily seizures. The reduced bursting observed, therefore, may reflect a more advanced pathological condition. Indeed, considerable cell death, spine loss, and reduced dendritic branching were observed in these animals [44].

Several studies have now demonstrated that the subiculum is a focus for the generation of spontaneous inter-ictal epileptiform activity in human patients [32, 45, 46]. Three factors have been proposed to contribute to this hyperexcitability: reduction of GABAergic inhibition or conversion to depolarizing inhibition [32, 42, 45–50], potentiation of recurrent excitatory connections within the subiculum [44], and reduced afterhyperpolarization following action potential firing [45]. It is possible that synaptic and non-synaptic changes interact and contribute to development of hyperexcitable network circuitry. For example, the enhanced bursting, that we and others observed, can potentiate recurrent excitation as bursting can lead to robust Hebbian synaptic plasticity in the hippocampus [51, 52].

Enhanced bursting behavior in the subiculum over time in the mature animals parallels the age dependence of the development of spontaneous recurrent seizures following KA-SE. Bursting cells have been shown to lead network bursts in a slice-based model of epilepsy [53], suggesting that enhanced bursting may lead to enhanced epileptiform activity in the hippocampus. The subiculum is the gateway to the rest of the brain, and specifically the neocortex [22–24]. Among its many extrinsic projections, the subiculum has extensive reciprocal connections with the entorhinal cortex, which is also known to be involved in temporal lobe epilepsy [54–58]. High frequency stimulation of subiculum can block focal seizures [59], while disinhibition has been shown to facilitate the spread of seizure activity from the subiculum to entorhinal cortex [41]; enhanced bursting, irrespective of specific proximal to distal spatial organization of bursting neurons [26, 60], would be expected to facilitate this effect. Thus, aberrant excitatory connections between subiculum and the entorhinal cortex, unchecked by normal GABAergic inhibition and limits on intrinsic bursting, could lead to the spread of seizures outside of the hippocampus, enabling the occurrence of generalized convulsive seizures.

## Supporting Information

S1 Fig Whole-cell current clamp recordings in response to square 600 ms-long currents. Typical responses to threshold currents (upper traces) and-200 pA (lower traces) are shown. SB neurons exhibited burst with two action potentials in response to threshold current at the onset of current injection, while RS neurons exhibited single action potential during threshold current injection. Hyperpolarizing pulses of currents induced sag.
(TIF)
