# Supplementary figures and images for: Age-Dependent Changes in Intrinsic Neuronal Excitability in Subiculum after Status Epilepticus
Source: PLoS One. 2015 Mar 16;10(3):e0119411. doi: 10.1371/journal.pone.0119411 (PMC4361192; doi:10.1371/journal.pone.0119411)

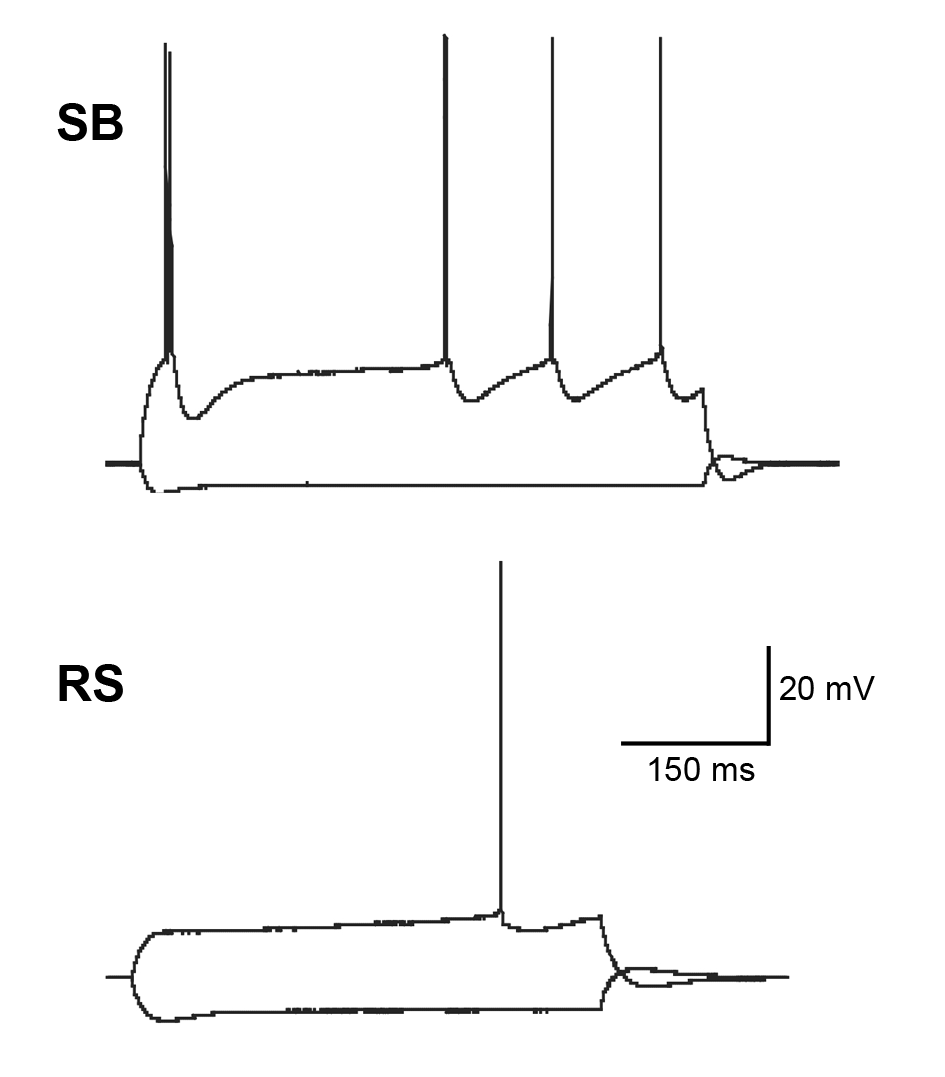

Supplement: S1 Fig — Typical responses to threshold currents (upper traces) and-200 pA (lower traces) are shown. SB neurons exhibited burst with two action potentials in response to threshold current at the onset of current injection, while RS neurons exhibited single action potential during threshold current injection. Hyperpolarizing pulses of currents induced sag. (TIF) [file pone.0119411.s001.tif]
